# Pelvic ultrasound and pubertal attainment in girls with sexual precocity: the pivotal role of uterine volume in predicting the timing of menarche

**DOI:** 10.3389/fendo.2024.1417281

**Published:** 2024-06-25

**Authors:** Alessandro Cattoni, Gianni Russo, Giulia Capitoli, Giulia Rodari, Maria Laura Nicolosi, Silvia Molinari, Daniele Tondelli, Ciretta Pelliccia, Silvia Radaelli, Andrea Mario Luciano Arosio, Katia Fontana, Giulia Tattesi, Paolo Passoni, Annalisa Boneschi, Claudia Giavoli, Silvia Laura Carla Meroni, Marianna Rita Stancampiano, Elda Garuti, Andrea Biondi, Adriana Balduzzi, Carla Bizzarri

**Affiliations:** ^1^ Department of Pediatrics, Fondazione IRCCS San Gerardo dei Tintori, Monza, Italy; ^2^ School of Medicine and Surgery, Milano-Bicocca University, Monza, Italy; ^3^ Department of Pediatrics, Endocrine Unit, IRCCS San Raffaele Scientific Institute, Milan, Italy; ^4^ Bicocca Bioinformatics, Biostatistics and Bioimaging B4 Centre, Department of Medicine and Surgery, University of Milano-Bicocca, Monza, Italy; ^5^ Department of Clinical Sciences and Community Health, University of Milan, Milan, Italy; ^6^ Endocrinology Unit, Fondazione IRCCS Ca’ Granda Ospedale Maggiore Policlinico, Milan, Italy; ^7^ Department of Pediatrics, Ospedale Papa Giovanni XXIII, Bergamo, Italy; ^8^ Department of Gynecology and Obstetrics, Fondazione IRCCS San Gerardo dei Tintori, Monza, Italy; ^9^ Department of Radiology, IRCCS San Raffaele Scientific Institute, Milan, Italy; ^10^ Unit of Pediatric Endocrinology, Ospedale Pediatrico Bambino Gesù, IRCCS, Rome, Italy

**Keywords:** precocious puberty, pelvic ultrasound, uterine volume, age of menarche, GnRH analogues

## Abstract

**Introduction:**

Among girls assessed for pubertal precocity, pelvic ultrasound (pUS) may represent a pivotal tool to predict the time expected to elapse between sonographic assessment and the onset of menarche (T_US-M_). Accordingly, the present analysis is meant to define the statistical relationship between sonographic parameters and T_US-M_, in order to identify the most reliable predictor of the timing of menarche.

**Methods:**

Retrospective, multicenter analysis. Girls assessed for sexual precocity and showing sonographic and clinical findings consistent with pubertal onset upon referral were considered eligible. Patients treated with GnRH analogues were excluded and only those who had subsequently achieved complete and spontaneous pubertal attainment and for whom the exact date of menarche was available were included. Overall, we enrolled 184 girls from five tertiary care Italian Centers.

**Results:**

The time elapsed (months) between baseline endocrine assessment and spontaneous achievement of menarche showed a negative statistically significant correlation (*p*<0.0001) with LH (r:-0.61), FSH (r:-0.59), estradiol (r:-0.52) and stimulated LH values (r:-0.58). Among pUS parameters, ovarian volume (r:-0.17 left, -0.30 right) and uterine body-to-cervix *ratio* (r:-0.18) poorly correlated with T_US-M_, while uterine diameters (r:-0.61 longitudinal, -0.64 anteroposterior) and volume (r:-0.70) achieved a highly statistical significance (*p*<0.0001). Uterine volume (UV) showed a negative logarithmic relationship with T_US-M_ and represented the most reliable predictor of the timing of menarche in uni- and multivariable analyses (*p <*0.001). ROC analyses identified the UV thresholds that best predict the onset of menarche within 18, 12 and 6 months, respectively: 3.76, 6.02 and 8.80 ml.

**Conclusion:**

The logarithm of UV shows the best statistical performance in predicting the timing of menarche in girls assessed for pubertal precocity. Accordingly, we developed a user-friendly online application that provides clinicians with an estimation of the months expected to elapse before menarche, based on the UV recorded upon pUS.

## 1 Introduction

The physiological timing of pubertal onset and subsequent attainment is under the control of a sophisticated regulatory network dynamically affected by a variety of endogenous and environmental factors (1). Several studies have outlined that genetic variables play a key role in this setting and affect 50% to 80% of the overall variation in the timing of puberty (2). On the other hand, exogenous variables including diet, body composition, chronic inflammatory disorders and endocrine-disrupting chemicals have been widely demonstrated to affect the timing and *tempo* of pubertal progression (3).

As a result of the synergistic effect of endogenous and exogenous factors, a growing body of literature has shed light on the progressive anticipation of pubertal attainment in Western Countries, where precocious puberty affects 1 in 5,000 to 10,000 children (4).

Accordingly, suspected disorders of the timing of pubertal attainment represent a remarkable share of all the referrals to tertiary care Pediatric Endocrine Centers.

In females, the differential diagnosis between central precocious puberty and benign pubertal variants (i.e. isolated precocious thelarche) leads to crucial prognostic and therapeutic implications and can be fulfilled only by performing an integrated evaluation of clinical, auxological, biochemical and radiological data (5).

In the last decades, pelvic ultrasound (pUS) has become a cornerstone in the diagnostic work-up of precocious or early puberty in girls. Several analyses have focused on the definition of the sonographic thresholds that support clinicians in discerning girls with para-physiological pubertal variants *versus* those for whom pubertal onset has already occurred. Uterine (volume, longitudinal diameter, body-to-cervix *ratio* and uterine arteries pulsatility index) and ovarian sonographic parameters (volume, follicle number and size) have been extensively assessed as potential markers of pubertal stimulation. Nevertheless, conflicting outcomes have been found and literature reports non-univocal cut-off values. In detail, proposed threshold levels range from 1.07 to 3.48 mL for uterine volume, from 22 to 37.4 mm for uterine longitudinal diameter and from 1.2 to 2.0 mL for ovarian volume (6–10). Furthermore, body-to-cervix *ratio* exceeding 0.8 to 1.0 has been identified as an additional sonographic parameter supporting the exposure of internal genitalia to sexual steroids (8, 9).

In patients showing clinical and biochemical signs consistent with central precocious or early puberty, the theoretical prediction of the timing of menarche plays a pivotal role in the decision-making process that eventually leads to the prescription of GnRH analogues. In this setting, pUS may be regarded as an informative tool to assess pubertal attainment and to provide a theoretical prediction of the timing of menarche. Accordingly, by retrospectively assessing the time elapsed between uterine and ovarian findings assessed by pUS and the onset of menarche in a multicentric cohort of girls evaluated for precocious or early puberty, we designed the present analysis to outline the statistical integrated relationship between sonographic findings over time (independent variables) and the estimated timing of menarche (dependent variable). We aimed at setting the theoretical cut-off points for each sonographic parameter, i.e. threshold values that allow clinicians to predict a defined outcome (occurrence of menarche within 6, 12 and 18 months following the pUS) with satisfactory sensitivity and specificity. As a result, we developed an informatic tool meant to dynamically estimate the expected onset of menarche based on the sonographic findings recorded upon pUS.

## 2 Materials and methods

### 2.1 Study design

A retrospective, multicenter, observational analysis took place in the following Italian Centers:

-Fondazione IRCSS San Gerardo dei Tintori, Monza.
-Ospedale Pediatrico Bambino Gesù, IRCCS, Rome.
-IRCCS Istituto Scientifico San Raffaele, Milan.
-Ospedale Papa Giovanni XXIII, Bergamo.
-Fondazione IRCCS Ca’ Granda Ospedale Maggiore Policlinico, Milan.

### 2.2 Patients’ eligibility criteria

Female patients, referred between 5.0 and 9.5 years due to precocious or early puberty and for whom clinical and/or biochemical signs consistent with pubertal activation were confirmed by a pediatric endocrinologist, were considered for eligibility. Eligible patients were included in the study only whether one or more of the following sonographic criteria, consistent with pubertal activation according to the most recent published literature (7, 8, 11–14), were fulfilled upon pUS: uterine volume ≥ 2 ml, uterine longitudinal diameter ≥ 35 mm, uterine body/cervix ratio > 0.9, endometrium thickness > 2 mm or ovarian volume ≥ 2 ml. All the sonographic exams were performed by skilled gynecologists and radiologists with proved expertise in the field of pubertal assessment. Finally, all eligible patients were included in the study only after they had achieved spontaneous menarche.

Exclusion criteria encompassed: administration of GnRH analogues to pharmacologically arrest pubertal progression; puberty induced pharmacologically; non-idiopathic precocious puberty; underlying demonstrated or suspected genetic disorders; recorded height upon enrollment <-3.0 SDS with reference to national growth charts, as severe short stature may be associated to an underestimation of uterine parameters; confirmed malformations of the uterine anatomy; previous history consistent with cranial or pelvic radiotherapy.

### 2.3 Data collection and source

The following variables were recorded: clinical information recorded upon examination (Tanner stage, degree of estrogenization), family history consistent with precocious or early puberty, anthropometric data (height SDS, weight SDS and height velocity SDS), basal biochemical values (LH, FSH, estradiol), stimulated hormonal levels following standardized GnRH stimulation test (LH and FSH peak, LH-to-FSH ratio), bone age assessed by non-dominant hand and wrist X-ray, uterine and ovarian parameters recorded by trans-abdominal pUS and the date of menarche.

Clinical, biochemical and radiological data were collected between September 2020 and September 2023 by a single operator at each Centre.

Clinical, auxological and anamnestic data were retrieved from patient’s electronic records. Raw auxological data (height, height velocity and weight) were converted into the corresponding SDS (standard deviation scores) values through a dedicated software (Growth® 4.0) with reference to the national (15) (height and weight) or international (Tanner charts, height velocity) growth charts (16).

Bone age was estimated by a single pediatric endocrinologist for each Centre, by the active re-reading of all the X-rays of non-dominant hand and wrists. All bone ages were estimated by Greulich and Pyle method.

Pelvic ultrasounds were performed in each Centre by gynecologists with a dedicated expertise in the field of female precocious puberty. The following parameters were collected: uterine diameters (mm), uterine volume (ml), body and uterine cervix and body transversal diameter (mm), uterine body-to-cervix ratio, endometrial line, if present (mm), right and left ovarian volume (ml), number of ovarian follicles and the maximum diameter of the largest follicle (mm). If not stated in the report, uterine and ovarian volumes were calculated from the recorded diameters according to the commonly used formula: uterine or ovarian volume (ml) = diameter 1 (mm) x diameter 2 (mm) x diameter 3 (mm) x 0.5233/1000.

The date of menarche, available for all enrolled patients, was recorded by consulting outpatient records. In selected cases, whenever it was not directly retrievable, the date of menarche was collected following direct telephone contact with caregivers. If the exact day of menarche was not available, a margin of variability of ± 15 days was considered acceptable.

### 2.4 Pelvic ultrasound

The sonographic evaluation was performed by trans-abdominal ultrasound, using a convex or microconvex probe (5–8MHz). By means of a median longitudinal scan, uterine cervix and body were assessed, including both the cervical canal and the endometrial cavity. By this approach, gynecologists managed to assess the longitudinal and anteroposterior diameters of the organ, along with cervix and body length. A transverse scan was used to assess uterine transverse diameter, ovarian and round ligaments and the endometrial rim. The ovaries were always visualized by both longitudinal and transverse scanning, measuring their three maximum diameters and relative volume.

Pelvic ultrasound, clinical evaluation, biochemical data and bone and wrist X-rays for bone age were performed at the same time for all the patients enrolled. Whenever the time elapsed between pUS and the assessment of the remaining clinical/biochemical/radiological evaluations exceeded ±1 month, patients were excluded from the present analysis.

### 2.5 Statistics

Continuous variables were reported as median and relative interquartile [Q1-Q3] range, while categorical variables were reported as count and frequency. The chi-square and Mann-Whitney tests were used to make comparisons between groups in terms of categorical and continuous variables, respectively.

The Pearson coefficient of correlation was calculated to quantify the degree of association between the sonographic parameters and the timing of the onset of menarche. A linear regression model was used to estimate their relationship. When the assumption of the linearity of the effect in continuous covariates was not met, the associations between the uterine sonographic parameters and the timing of the onset of menarche were explored through linear models in the natural logarithm of the predictor variable to mimic an exponential decaying model. Univariate logistic models were used to assess the impact of several sonographic or biochemical parameters on the outcome (possibility of onset of menarche by 12 or 18 months). Receiver Operating Characteristic (ROC) curves were used to define, for each sonographic parameter, the best cut-off point to predict the occurrence of menarche by a defined time interval (6, 12 or 18 months) following sonographic evaluation. For each ROC curve, the AUC (Area Under the Curve) was defined as a measure of the diagnostic performance of the identified best cut-off points. Kaplan–Meier method was used to compare the survival curves of different groups (i.e., subcohorts of patients defined by the cut-off point of the sonographic parameter). Finally, univariate regression models were used to assess the predicted role of various factors on the timing of the onset of menarche. Multivariable regression model was performed by including the most clinically meaningful independent variables within the set of those significant at the univariate analysis based on the value of the Akaike information criterion.

The tests performed were 2-sided, and the significance level was set as p < 0.05. All statistical analyses were performed using open-source R software v.4.4.2 (R Foundation for Statistical Computing, Vienna, Austria).

## 3 Results

### 3.1 Clinical and demographic features

By applying a stepwise selection process, 184 girls fulfilling the selection criteria were identified. One or more pUS, performed to assess internal genitalia in the setting of early or precocious puberty, were available for all the patients enrolled.

From a clinical perspective, the reported median age upon the onset of the first signs consistent with pubertal onset was 7.48 (IQR: 6.81–8.13) years. The patients enrolled underwent pUS at the average age of 8.25 (IQR: 7.69–8.82) years, while they were aged 10.29 (IQR: 9.79–10.81) years when they achieved spontaneous menarche. Accordingly, the average time elapsed between pUS and menarche (T_US-M_ *)* was 23 (IQR: 15–31) months.

Overall, T_US-M_ was < 6.0 months in 7.1% (n=13) of patients, between 6.0 and 11.9 months in 8.2% (n=15), between 12 and 17.9 months in 15.2% (n=28) and ≥ 18.0 months in the remaining 69.6% (n=128).

The distribution of Tanner stages with reference to the time elapsed between the endocrine evaluation and the onset of menarche is shown in **Table 1**. We found that Tanner stage for breast (B) and pubic hair (PH) was statistically associated (*p <*0.001) with the timing of menarche with reference to all the *cut-off* established (6, 12 and 18 months). On the other hand, Tanner staging for axillary hair (AH) was statistically more advanced among patients who achieved menarche by 6 months compared to those who achieved menarche later (*p* 0.008), whereas no statistically significant association could be retrieved for the remaining thresholds assessed (*p* 0.207 and 0.183 for 12 and 18 months, respectively).

**Table 1:** Distribution of Tanner stages, biochemical findings and bone age with reference to the time elapsed between the clinical/sonographic/biochemical evaluation and the onset of menarche (T_US-M_).

Clinical parameter	Tanner stage	N of patients	T_US-M_ ≥ 6.0 months	T_US-M <_6.0 months	*p value*	T_US-M_ ≥ 12.0 months	T_US-M <_12 months	*p value*	T_US-M_ ≥18.0 months	T_US-M <_18 months	*p value*
		184	171	13		156	28		128	56	
Breast	B2	117 (63.6%)	117 (68.4%)	0 (0.0%)	<0.001	112 (71.8%)	5 (17.9%)	<0.001	98 (76.6%)	19 (33.9%)	<0.001
	B3	62 (33.7%)	52 (30.4%)	10 (76.9%)		44 (28.2%)	18 (62.3%)		30 (23.4%)	32 (57.1%)	
	B4	5 (2.7%)	2 (1.2%)	3 (23.1%)		0 (0.0%)	5 (17.8%)		0 (0.0%)	5 (8.9%)	
Pubic hair	PH1	65 (35.3%)	65 (38.0%)	0 (0.0%)	<0.001	62 (39.7%)	3 (10.7%)	<0.001	52 (40.6%)	13 (23.2%)	0.001
	PH2	80 (43.5%)	75 (43.9%)	5 (38.5%)		71 (45.5%)	9 (32.1%)		57 (44.5%)	23 (41.1%)	
	PH3	32 (17.4%)	27 (15.8%)	5 (38.5%)		21 (13.5%)	11 (39.3%)		18 (14.1%)	14 (25.0%)	
	PH4	7 (3.8%)	4 (2.3%)	3 (23.0%)		2 (1.3%)	5 (17.9%)		1 (0.8%)	6 (10.7%)	
Axillary hair	AH1	116 (63.1%)	112 (65.5%)	4 (30.8%)	0.008	102 (65.4%)	14 (46.2%)	0.207	86 (67.2%)	30 (53.6%)	0.183
	AH2	63 (34.2%)	56 (32.7%)	7 (53.8%)		50 (32.0%)	13 (50.0%)		39 (30.5%)	24 (42.8%)	
	AH3	5 (2.7%)	3 (1.8%)	2 (15.4%)		4 (2.6%)	1 (3.8%)		3 (2.3%)	2 (3.6%)	

Biochemical parameter		N of patients with available data	T_US-M_ ≥ 6.0 months	T_US-M_ <6.0 months	*p value*	T_US-M_ ≥ 12.0 months	T_US-M_ <12 months	*p value*	T_US-M_ ≥18.0 months	T_US-M_ <18 months	*p value*
LH (U/L)		134	0.3 (0.1–0.4)	4.7 (4.4–5.2)	<0.001	0.3 (0.1–0.4)	4.2 (2.4–4.9)	<0.001	0.3 (0.1–0.3)	2.2 (0.3–4.2)	<0.001
FSH (U/L)		132	2.6 (1.6–4.2)	6.3 (6.0–7.7)	<0.001	2.4 (1.5–3.8)	7.1 (5.6–8.1)	<0.001	2.4 (1.3–3.5)	5.2 (2.9–7.3)	<0.001
Estradiol (pg/ml)		134	14.7 (5.0–27.9)	45.0 (40.5–66.0)	<0.001	13.1 (5.0–25.3)	39.2 (28–53)	<0.001	12.4 (5.0–23.1)	26.6 (17.5–40.9)	<0.001
LH peak following GnRH (U/L)		106	4.1 (2.3–10.8)	34.5 (28.2–46.7)	<0.001	3.9 (2.2–9.7)	22.9 (10.1–29.6)	<0.001	3.7 (2.1–7.9)	15.5 (7.6–26.1)	<0.001

Radiological parameter		N of patients with available data	T_US-M_ ≥ 6.0 months	T_US-M_ <6.0 months	*p value*	T_US-M_ ≥ 12.0 months	T_US-M_ <12 months	*p value*	T_US-M_ ≥18.0 months	T_US-M_ <18 months	*p value*
Difference between bone age and chronological age (years)		154	1.0 (0.4–1.8)	0.85 (0.6–1.5)	0.225	0.91 (0.3- 1.7)	1.7 (0.8–2.00)	0.076	0.91 (0.4–1.6)	1.35 (0.4–1.9)	0.371

Patients are classified with reference to three different T_US-M_ thresholds: 6, 12 and 18 months. Qualitative variables (i.e. the distribution of clinical parameters with reference to T_US-M_ classes) were assessed by Fisher’s exact test. Conversely, median biochemical and sonographic parameters (continuous variables) among patients who achieved menarche before or after a definite threshold were compared through Mann-Whitney U test.

### 3.2 Biochemical findings: distribution and correlation with time-to-menarche

From a biochemical perspective, as showed in **Table 1**, unstimulated LH, FSH, estradiol and LH peak following GnRH administration assessed upon pUS were statistically greater (*p*<0.001) among patients who achieved menarche by 6, 12 and 18 months compared to girls for whom T_US-M_ exceeded these time thresholds.

In addition, time-to-menarche showed a negative statistically significant correlation with LH (r: -0.61, *p <*0.0001), FSH (r: -0.59, *p <*0.0001) and estradiol values (r: -0.52, *p <*0.0001) recorded upon pUS. A superimposable statistical relationship was retrieved between T_US-M_ and LH peak values achieved following standardized GnRH stimulus (r: -0.58, *p <*0.0001).

**Figure 1:**
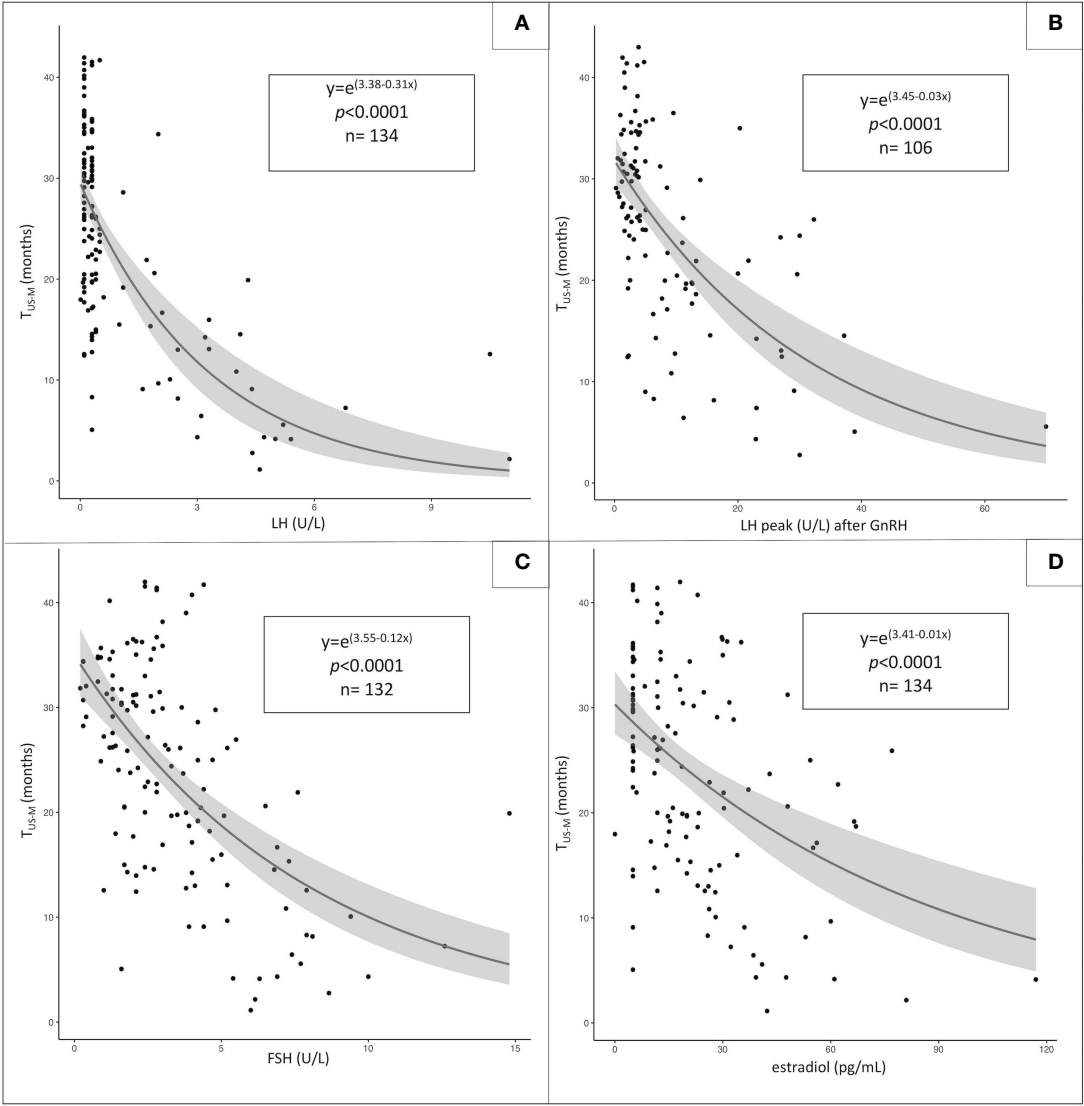
Statistical relationship between the time elapsed between sonographic/biochemical assessment and menarche (T_US-M_, dependent variable) and the following independent variables: unstimulated LH **(A)**, LH peak following GnRH administration **(B)**, unstimulated FSH **(C)** and estradiol values **(D)**. The most-fitting correlation statistical pattern is logarithmic for all the independent variables assessed.

As reported in **Figure 1**, the statistical function that provides the most accurate estimation of the relationship between T_US-M_ (dependent variable) and each of the abovementioned biochemical parameters (independent variables) is logarithmic.

### 3.3 Sonographic parameters: distribution and correlation with time-to-menarche

The median value and IQR range of the sonographic parameters collected in the whole study population and the relative distribution with reference to the time-to-menarche are reported in **Table 2**.

**Table 2:** Distribution of uterine and ovarian parameters assessed by pelvic ultrasound with reference to the time elapsed between the sonographic evaluation and the onset of menarche (T_US-M_).

Sonographic parameters	Number of patients	Whole study population	T_US-M_ ≥ 6 months	T_US-M_ < 6 months	*p value*	T_US-M_ ≥ 12 months	T_US-M_ < 12 months	*p value*	T_US-M_ ≥ 18 months	T_US-M_ < 18 months	*p value*
Uterine volume (mL)	140	2.9 (1.8–5.5)	2.7 (1.7–4.6)	14.0 (10.4–16.1)	<0.001	2.5 (1.7–3.5)	10.2 (7.1–13.8)	<0.001	2.5 (1.60–3.19)	7.4 (4.9–10.2)	<0.001
Uterine longitudinal diameter (mm)	136	38.0 (33.0–43.0)	38.0 (33.0–42.0)	50.0 (45.0–64.5)	<0.001	37.0 (32.0–40.0)	50.0 (45.0–54.0)	<0.001	36.0 (31.0–39.0)	45.0 (41.0–50.8)	<0.001
Uterine transverse diameter (mm)	116	15.0 (11.4–20.0)	15.0 (11.0–19.0)	30.0 (30.0–32.0)	<0.001	14.0 (11.0–17.0)	24.5 (22.0–30.0)	<0.001	13.0 (11.0–17.0)	22.0 (16.0–26.0)	<0.001
Uterine antero-posterior diameter (mm)	112	10.0 (8.0–13.0)	9.0 (7.8–12.0)	18.0 (18.0–23.0)	<0.001	9.0 (7.3–11.8)	17.0 (14.3–19.0)	<0.001	9.0 (7.0–11.0)	14.0 (12.0–18.0)	<0.001
Uterine body-to-cervix ratio	67	1.1 (1.0–1.5)	1.1 (1.0–1.4)	1.7 (1.4–2.0)	0.058	1.1 (1.0–1.3)	1.6 (1.2–1.8)	0.005	1.1 (1.0–1.3)	1.5 (1.1–1.8)	0.002
Left ovarian volume (ml)	135	2.0 (1.4–2.8)	1.9 (1.4–2.7)	2.8 (2.0–5.1)	0.032	1.9 (1.3–2.6)	2.5 (1.8–3.7)	0.024	1.8 (1.4–2.6)	2.2 (1.4–3.0)	0.156
Right ovarian volume (ml)	135	2.2 (1.4–3.2)	2.1 (1.4–2.9)	3.8 (2.9–4.9)	0.008	2.0 (1.4–2.9)	3.4 (2.20–4.60)	0.004	2.0 (1.4–2.8)	2.6 (1.9–4.0)	0.006
Diameter of the maximum ovarian follicle (mm)	90	5.8 (4.7–7.4)	5.0 (4.7–7.0)	7.0 (6.1–8.6)	0.121	5.0 (4.4–7.0)	6.8 (5.3–8.0)	0.03	5.0 (4.0–7.0)	6.0 (5.0–8.0)	0.031

All the parameters are reported as median value and relative interquartile range (IQR). Patients are classified with reference to three different T_US-M_ thresholds: 6, 12 and 18 months.

Uterine volume and diameters and right ovarian volume were statistically greater among patients who achieved menarche by 6, 12 and 18 months compared to the girls for whom T_US-M_ exceeded these time thresholds. Conversely, left ovarian volume, uterine body-to-cervix ratio and the diameter of the greatest ovarian follicle showed a statistically significant association with time-to-menarche only with reference to some of the thresholds considered, often with borderline significance (see **Table 2**).

**Figure 2:**
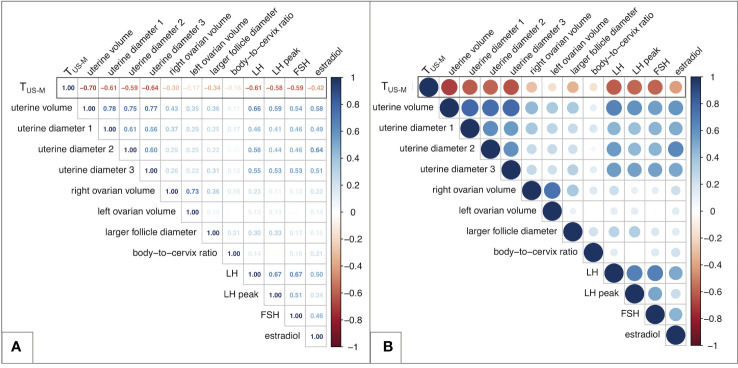
Matrix of the correlation coefficients between couples of biochemical and/or sonographic variables. **(A)** reports the Pearson’s coefficients for all the couples of variables. **(B)** displays a graphical equivalence for each correlation, represented as a filled circle. The greater the coefficient, the more intense is the color of each circle, with red representing negative correlations and blue positive ones. In addition, the diameter of each circle shows an inverse relationship with the *p value* of the specific correlation assessed. Overall, larger and darker circles represent more statistically significant and strong correlations, while transparent and small-sized circles indicate weaker and poorly statistically significant relationships between the variables assessed. T_US-M_ – time elapsed between ultrasound/biochemical evaluation and menarche.

A graphical representation of all the coefficients recorded when assessing the statistical correlation between couples of biochemical and sonographic variables is reported as a matrix in **Figure 2**. Among the latter, uterine volume (r: -0.70; *p <*0.0001), longitudinal and anteroposterior uterine diameters (r_long_: -0.61, r_AP_: -0.64; *p <*0.0001) presented with the strongest and most fitting negative correlation with T_US-M_ and were therefore included in subsequent analyses. Conversely, body-to-cervix ratio (r: -0.18; *p* 0.2) as well as left (r: -0.17; *p* 0.042) and right (r: -0.30; *p* 0.0004) ovarian volume and the diameter of the largest ovarian follicle (r: -0.34; *p* 0.001) presented with an unsatisfactory correlation with T_US-M_.

**Figure 3:**
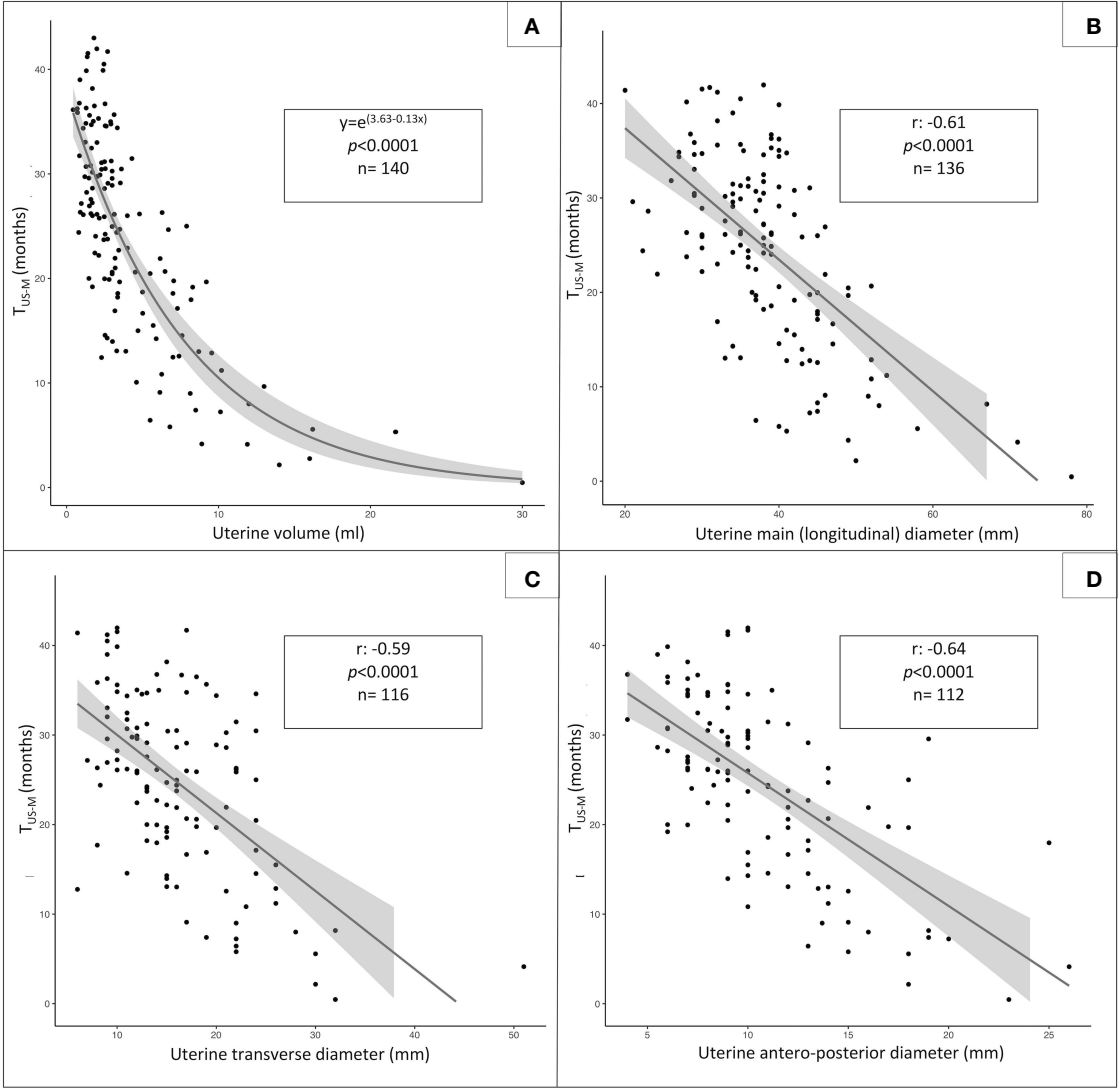
Statistical relationship between T_US-M_, (time elapsed from pelvic ultrasound and menarche, dependent variable) and the following sonographic parameters: uterine volume (**A**, logarithmic function), uterine longitudinal diameter (**B**, linear function), uterine transversal diameter (**C**, linear function) and anteroposterior diameter (**D**, linear function).

The distribution of the uterine sonographic parameters (independent variables) plotted against T_US-M_ (dependent variable) is represented in **Figure 3**. While the correlation that provides the best description of the relationship between uterine diameters and T_US-M_ is linear, the most fitting statistical function for uterine volume is logarithmic. This trendline is confirmed by the improvement in the AIC index when uterine volume is reported as a logarithmic rather than linear model upon univariable analysis (**Table 3**).

**Table 3:** Univariate analysis of the impact of different sonographic and biochemical parameters on the timing-to-menarche.

Variable	AIC	coefficient	*p* value	CI low	CI high
Uterine volume	952.53	-1.718	<0.001	-2.007	-1.429
Log (uterine volume)	929.346	-9.861	<0.001	-11.283	-8.44
Uterine longitudinal diameter	953.28	-0.696	<0.001	-0.849	-0.544
Log (longitudinal diameter)	959.069	-26.858	<0.001	-33.088	-20.628
Uterine anteroposterior diameter	775.888	-1.485	<0.001	-1.815	-1.154
Log (anteroposterior diameter)	778.146	-16.82	<0.001	-20.658	-12.981
LH	949.056	-3.431	<0.001	-4.188	-2.675
LH stimulated peak (continuous variable)	743.514	-0.512	<0.001	-0.651	-0.372
LH stimulated peak (dicotomic variable)	1374.936	-5.897	<0.001	-9.158	-2.637
Age	1317.49	-6.227	<0.001	-7.56	-4.894

The variables included in the present analysis are those displaying the most satisfactory statistical correlation with the time-to-menarche. When comparing linear and logarithmic models for each variable, a lower AIC was associated to a more fitting statistical relationship with the time-to-menarche. AIC, Aikake’s information criterion; CI, confidence interval.

### 3.4 Uterine parameters: definition of best cut-off points in predicting the timing of menarche

**Figure 4:**
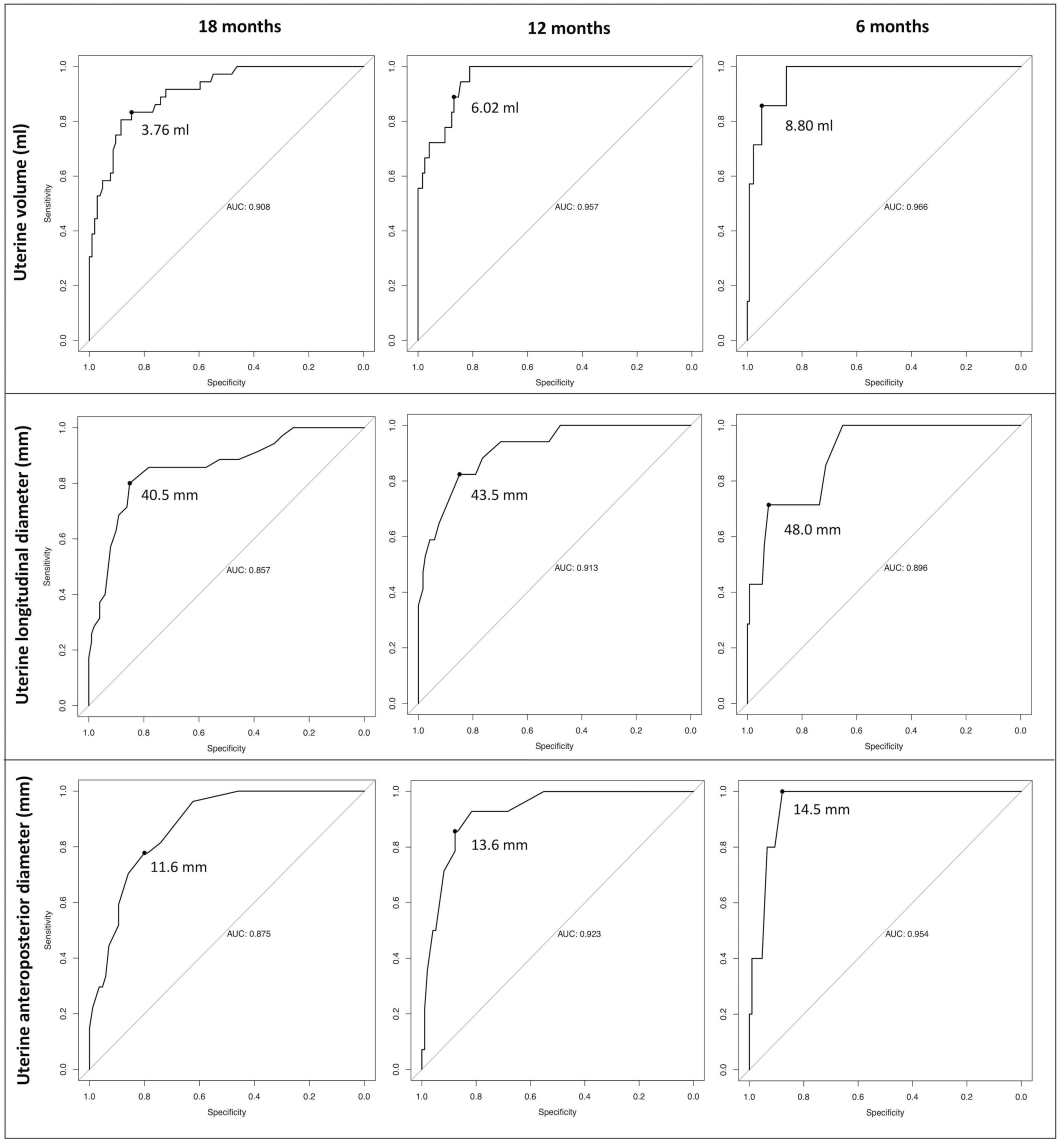
Receiver operator characteristic (ROC) curves designed to define the *best cut-off points* for the three uterine sonographic parameters assessed (uterine volume, longitudinal and anteroposterior diameters). Accordingly, we defined the threshold values that display the best statistical accuracy in predicting the onset of menarche by pre-defined time intervals (6,12 and 18 months). Patients with uterine parameters exceeding these thresholds are expected to achieve menarche within the corresponding time span with the most satisfactory combination of sensitivity and specificity.

By running dedicated ROC curves (**Figure 4**), we identified the threshold values of uterine volume and diameters that showed the best statistical accuracy in predicting the onset on menarche by a specific time interval (6, 12 or 18 months). Accordingly, patients presenting with uterine parameters exceeding these *best cut-off points* are expected to achieve menarche within the corresponding time span with the most satisfactory combination of sensitivity and specificity.

For uterine volume, the *best cut-off points* were 3.76, 6.02 and 8.8 ml at 18, 12 and 6 months, respectively. Consistently, the 18, 12 and 6 months-thresholds were 40.5, 43.5 and 48 mm for the longitudinal uterine diameter and 11.6, 13.6 and 14.5 mm for the anteroposterior one.

The best cut-off points and relative AUC, sensitivity and specificity are summarized in **Table 4**.

**Table 4:** Threshold values for uterine volume, longitudinal and antero-posterior diameters that show the best statistical accuracy in predicting the onset on menarche by a specific time interval (6, 12 or 18 months).

Uterine parameter	Time threshold (months)	Best cut-off point	AUC	Sensitivity	Specificity
Uterine volume	18	3.76 ml	0.908	0.83	0.85
	12	6.02 ml	0.957	0.89	0.87
	6	8.80 ml	0.966	0.86	0.95
Uterine longitudinal diameter	18	40.5 mm	0.857	0.80	0.85
	12	43.5 mm	0.913	0.82	0.85
	6	48 mm	0.896	0.71	0.92
Uterine anteroposterior diameter	18	11.6 mm	0.875	0.78	0.80
	12	13.6 mm	0.923	0.86	0.88
	6	14.5 mm	0.954	1.00	0.88

Patients presenting with uterine parameters exceeding these best cut-off points are expected to achieve menarche within the corresponding time span with the most satisfactory combination of sensitivity and specificity.

AUC, area under the curve assessed by dedicated ROC (Receiver operating characteristic) curves.

**Figure 5:**
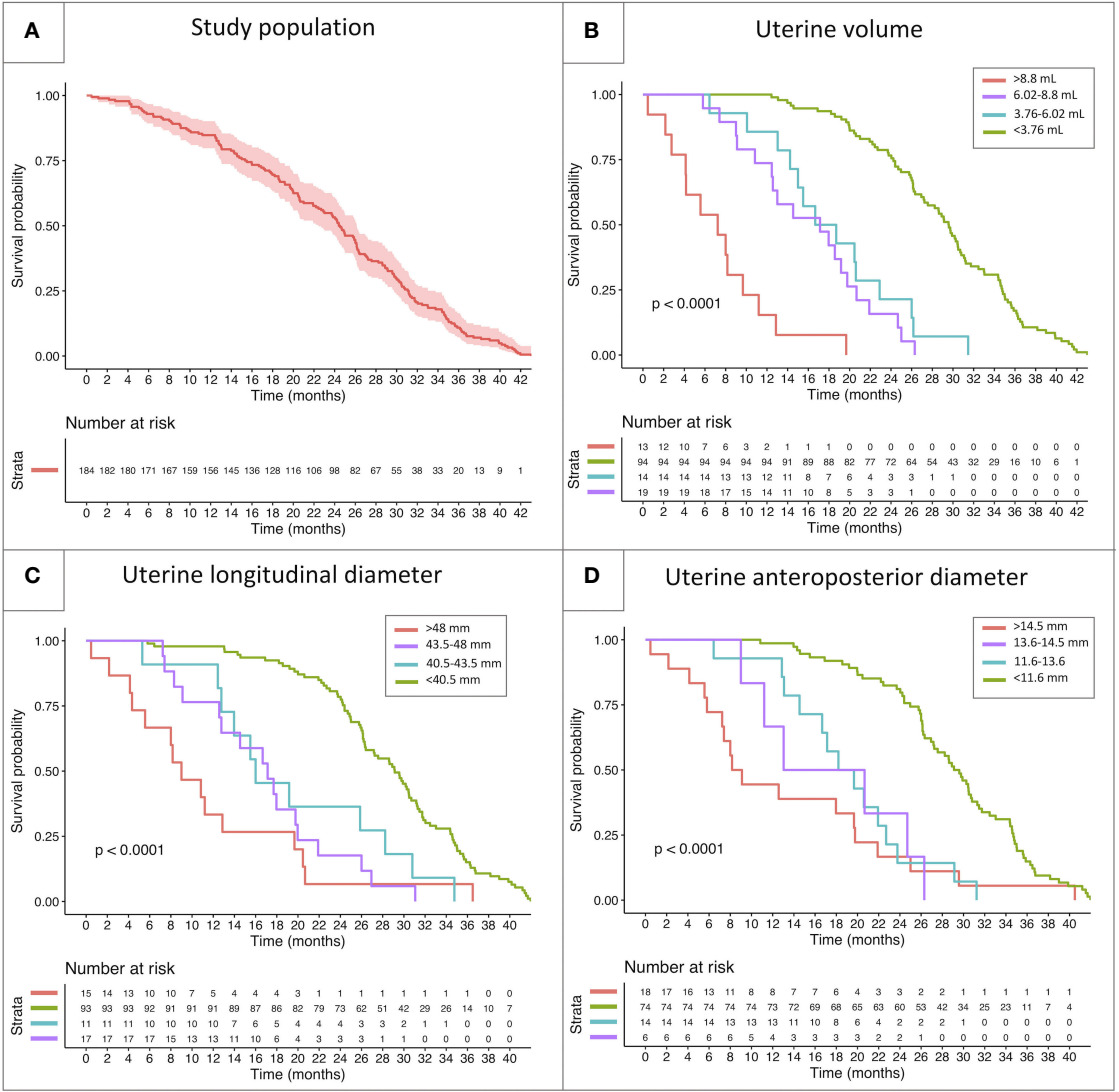
Kaplan-Meier model reporting the survivorship trendlines (event: onset of menarche) in the whole study population **(A)** and in specific subcohorts of patients classified with reference to the threshold values identified by the ROC curves reported above. The survivorship curves report the biological behavior of subclasses of patients showing different uterine volume **(B)**, longitudinal uterine diameter **(C)** and anteroposterior diameter **(D)**.

Based on the above-mentioned thresholds, the study population was subdivided into four categories, with reference to each parameter: below lower threshold, between lower and intermediate threshold, between intermediate and higher threshold and above upper threshold. The four categories showed a statistically different behavior for all the sonographic parameters assessed (uterine volume, longitudinal diameter and anteroposterior diameter, *p*<0.001) upon Kaplan-Meier models. While a certain degree of overlap in the survivorship trends could be noted for uterine diameters, the cut-offs identified for uterine volume successfully managed to discriminate the biological behavior of all the four subclasses (**Figure 5**).

### 3.5 Uterine volume as a pivotal determinant of time-to-menarche: from multivariable analysis to the development of a dedicated web tool

Upon multivariable analysis, uterine volume, expressed as a logarithmic variable, was the only sonographic parameter that achieved a statistically significant impact on the timing of menarche (**Table 5**, model 1 - *p* 0.0147). Conversely, neither uterine diameters nor stimulated LH played a statistically significant effect on T_US-M_.

**Table 5:** Multivariable analysis of the integrated impact of sonographic, biochemical and demographic data on the time-to-menarche.

Model 1	Estimate	SE	T value	*p* value
Intercept	41.8959	4.0395	10.371	<0.001
Log (uterine volume)	-6.1434	2.4772	-2.480	0.0147
Uterine longitudinal diameter	-0.1303	0.1012	-1.288	0.2007
Uterine transverse diameter	-0.1224	0.1768	-0.692	0.4904
Uterine anteroposterior diameter	-0.2631	0.2852	-0.923	0.3583
LH stimulated peak (dicotomic variable)	-2.0534	1.4219	1.444	0.1517

Model 2	Estimate	SE	T value	*p* value
Intercept	55.5203	5.3123	10.451	<0.001
Log (uterine volume)	-7.9204	0.8373	-9.459	<0.001
Age	-2.6119	0.6808	-3.836	<0.001
LH stimulated peak (dicotomic variable)	-2.0810	1.1881	-1.751	0.08212

SE, standard error.

The impact of uterine volume was retained also when age was included in the multivariable model (**Table 5**, model 2 – *p <*0.001).

Given the demonstrated pivotal role of uterine volume in predicting the timing of menarche, we finally developed a calculator that provides clinicians with an estimation of the months expected to elapse before menarche, based on the uterine parameters recorded. The tool is available online at:

https://b4-uni25-5627493duksfy852qr80fewbsn3986g43jkgkzie8.shinyapps.io/ECO-PUB/.

## 4 Discussion

The scientific community has widely acknowledged the undisputed auxological and psychological beneficial effects of early recognition and treatment of progressive precocious puberty among girls aged 6 years or younger. Conversely, patients for whom the onset of puberty is detected between 6 and 8 years deserve an individualized approach, and the prescription of GnRH analogues should be regarded as the output of an integrated evaluation of clinical, auxological, biochemical and radiological data (17, 18).

In this setting, as conflicting outcomes have been reported in terms of treatment-related estimated height gain, psychological issues related to pubertal changes play a pivotal role in the decision to delay menarche by prescribing GnRH analogues (19). Accordingly, providing clinicians and caregivers with an estimation of the time expected to elapse from endocrine assessment to the occurrence of menarche may represent a supportive element in the decision-making process that eventually leads to treatment prescription for patients aged 6 to 8 years upon reported onset of puberty.

Published evidence suggests that the average time elapsing between the onset of the earliest clinical markers consistent with pubertal onset (thelarche) and the achievement of spontaneous menarche ranges from 2 to 2.5 years (20). Nevertheless, it is not infrequent that patients are already well established into puberty upon the time of specialistic referral, and the retrospective collection of the timing of the onset and *tempo* of progression often leads to inaccurate estimations. In addition, while clinical, biochemical and sonographic thresholds that discern the onset of early puberty from benign pubertal variants have been extensively studied and are routinely assessed in clinical practice, the *tempo* of pubertal progression has been only rarely considered. Accordingly, to the best of our knowledge no research focused on the determinants of the time-to-menarche have been published to date.

In order to fulfill this gap of knowledge, we designed the present analysis, aiming at defining the theoretical relationship between clinical, biochemical and sonographic predictors (independent variables) and the time-to-menarche (dependent variable) in a population of girls assessed for idiopathic central precocious or early puberty.

The study population was retrieved by applying a stepwise selection process out of hundreds of girls assessed for suspected pubertal precocity in 5 Italian tertiary care Centers of pediatric endocrinology. As the time elapsed between clinical/biochemical/sonographic evaluation and the date of menarche was the main outcome of our analyses, we established stringent enrollment criteria, in order to exclude all potential biases affecting the spontaneous progression of puberty. Accordingly, patients treated with GnRH analogues were excluded from the analysis. Moreover, patients without biochemical/sonographic confirmation of pubertal activation, i.e. girls classified as presenting with pubertal variants were excepted. The inclusion of these latter would have resulted in an overestimation of the time-to-menarche and in the lack of a systematic statistical correlation with the variables assessed. As patients diagnosed with central precocious puberty at a younger age are more likely to be started on treatment with GnRH treatment *tout-court*, the median age upon reported onset of puberty in the selected population (7.48 years, IQR: 6.81–8.13) mirrors the class of patients that benefit the most from the clinical outcomes of the present analysis. Indeed, while patients younger than 6 years, mostly excluded from the present analysis, are candidate to therapy irrespectively of the time-to-menarche, a systematic prediction of this variable potentially represents a key element in tailoring a dedicated treatment plan in patients aged 6 to 8 years upon pubertal onset, with regard to psychological and cognitive background.

Patients as old as 9.5 years were included in the study. These latter were late referrals for marginally early pubertal development and showed incipient spontaneous menarche soon after the first clinical and sonographic evaluation. The inclusion of late referrals with advanced pubertal clinical ad sonographic findings was meant to collect information about the predictive role of greater US volumes/diameter on T_US-M_.

From a clinical perspective, as expected, more advanced Tanner stage for pubarche and thelarche, along with a greater degree of estrogenization, showed a statistically significant negative association with the time-to-menarche. Conversely, the prognostic role of axillary hair achieved statistical significance only in discerning girls with expected menarche by 6 months following the endocrine evaluation.

Biochemical endocrine data provide additional prediction over the expected time-to-menarche and we outlined a logarithmic trendline when plotting LH, FSH, estradiol and stimulated LH levels against the time elapsed between baseline assessment and menarche. Nevertheless, despite a statistically significant negative correlation for all the above-mentioned variables, none of them achieved a statistical significance upon multivariable analysis and all biochemical data displayed a markedly scattered distribution. The systematic analysis of the distribution of unstimulated LH values in our study population highlighted the dramatic variability of the time-to-menarche among patients with luteinizing hormone < 1 U/L, ranging from 5 to over 40 months. As baseline LH levels increase, the statistical relationship with the time expected to elapse until menarche achieve a more reproducible and fitting negative correlation.

Along with low unstimulated LH levels, the distribution of estradiol levels showed the most unsatisfactory statistical correlation with time-to-menarche. These outcomes are consistent with the growing body of literature that demonstrates the insufficient sensitivity and specificity of estradiol levels, especially < 30 pg/mL, in tracking the onset and progression of puberty (21, 22). Accordingly, as estradiol levels do not systematically mirror longitudinal pubertal attainment over time, a cross-sectional assessment cannot provide a reliable prediction of the time needed to achieve complete pubertal maturation.

In addition, inter-assay discrepancies in the lower detection thresholds of basal LH/FSH and estradiol values may have played a role in affecting the statistical relationship between these biochemical parameters and T_US-M_.

Over the last decades, pelvic ultrasound has been extensively recognized as an essential cornerstone in the diagnostic work-up of precocious puberty in females (12, 23). Indeed, several authors have identified sonographic thresholds that support clinicians in discerning cases consistent with precocious puberty from non-progressing benign pubertal variants. In addition, the longitudinal assessment of ovarian and uterine diameters and volumes over time represents a valuable tool to monitor the efficacy of GnRH analogues among treated patients (11).

By assessing sonographic parameters from a different perspective, we managed to demonstrate the key role of pelvic ultrasound also in providing clinicians with a reliable prediction of the timing of menarche among girls assessed for early or precocious puberty.

Ovarian parameters (right and left ovarian volume and number of follicles) showed an unsatisfactory statistical correlation with expected time-to-menarche, with Pearson’s coefficients (r) as low as -0.3, -0.17 and -0.34, respectively. Among uterine sonographic parameters, body-to-cervix *ratio* displayed the poorest statistical performance in assessing pubertal changes over time and in estimating the time expected to elapse before menarche. Despite its universally acknowledged role in promptly identifying pubertal onset (13, 24), we hypothesize that the theoretical explanation for a poor accuracy in predicting menarche can be retrieved in the mathematical nature of the measure itself. Indeed, the consensual increase in body (numerator) and cervix (denominator) diameters as puberty progresses hinders and flattens the longitudinal changes of the *ratio* that should track pubertal progression and mark incoming menarche.

Conversely, our data demonstrated that time-to-menarche showed a satisfactory correlation with uterine diameters and volume, with the latter representing the most predictive and reliable parameter among all the clinical, biochemical and sonographic variables assessed both at univariate and multivariable analyses. In detail, the distribution of uterine volume showed a highly significant negative logarithmic correlation with the time-to-menarche, and the logarithm of uterine volume was the only variable that achieved a statistical significance in a multivariable model. Though also uterine diameters showed a statistically significant negative correlation with time-to-menarche *per se*, it is likely that each mono-dimensional model less effectively mirrors the complex tridimensional uterine changes occurring over puberty, more fittingly embodied by an estimation of uterine volume. In addition, as ultrasound is operator-dependent, we hypothesize that minimal over- or underestimation of each single linear measurement are potentially smoothed by the tridimensional integration of the two remaining diameters, thus providing an ultimately more predictive sonographic parameter.

In order to provide clinicians with a practical application of the mathematical relationships outlined, we firstly identified the thresholds that achieve the best statistical accuracy in predicting the onset menarche by defined time intervals (6, 12 and 18 months), as reported in **Table 4**. By comparing sonographic findings with the thresholds hereby reported, we believe that pediatric endocrinologists and gynecologists involved in the diagnosis and treatment of precocious puberty may be supported in the decision-making process that eventually leads to GnRH prescription. Ultimately, we developed an interactive user-friendly web tool, meant to provide clinicians with an estimation of the months expected to elapse before menarche, based on the outcomes on uterine volume drawn in the present study. It is worthy noticing that the statistical methods implied for the definition of the abovementioned thresholds and for the development of this applicative are diverse, thus providing a different sort of information. Accordingly, a certain degree of discrepancy is expected when comparing the prediction of time to menarche assessed by the cut off points reported in **Table 4** or by using the web tool.

The innovative perspective and practical approach of our research represent its main strengths. Indeed, we believe that the setting of clinically-driven thresholds and the development of a user-friendly online application may guide pediatric endocrinologists in establishing the best therapeutic approach for girls already well settled into puberty upon referral.

In addition, in the light of the stringent enrollment criteria adopted, we managed to gather an overall wide population of homogeneous patients, that could be achieved only by performing a multicentric study.

On the other hand, the retrospective nature of the analysis represents a potential limitation. Firstly, for those patients for whom the exact date of menarche was not retrievable from medical records, it was gathered following phone consultation with patients’ guardians. Nevertheless, for most patients this information had been properly recorded on medical records and whenever caregivers showed any degree of hesitation about the exact date, the patient was excluded from the analysis. In addition, 82.6% of patients (152 out of 184) experienced menarche from 2018 onwards. Accordingly, as only few years had elapsed before the timing of data collection (2020–2022), the exact date could be easily retrieved upon phone consultation.

Another potential limitation is represented by the fact that sonography is an operator-dependent technique, which may result in a poorer reproducibility of the outcomes drawn. Nevertheless, a single skilled operator, with a specific commitment in pediatrics and with a long-standing expertise in the field, performed ultrasound evaluations in each of the for each of the 5 Centers. This highlights the importance of referring patients with a clinical suspicion of precocious puberty to gynecologists/radiologists with specific skills in the sonographic assessment of pubertal progression.

Finally, an additional study is warranted to validate the clinical accuracy and real-life reproducibility of the webtool developed though the present analysis.

## 5 Conclusions

By assessing the statistical relationship between clinical/biochemical/sonographic parameters and the time elapsed between endocrine assessment and menarche, we firstly highlighted that uterine volume, assessed by pelvic ultrasound, represents the most reliable and reproducible predictor of the expected time-to-menarche in a population of girls assessed for early or precocious puberty.

Accordingly, we outlined the dimensional thresholds for uterine diameters and volume that provide the best statistical accuracy in predicting the onset of menarche by pre-established time intervals (namely 6,12 and 18 months).

Finally, based on the outcomes gathered in the present analysis, we developed a user-friendly informatic tool that provides clinicians with a prediction of the expected time-to-menarche from uterine volume/diameters collected at pelvic ultrasound, available at:

https://b4-uni25-5627493duksfy852qr80fewbsn3986g43jkgkzie8.shinyapps.io/ECO-PUB/.

## Data availability statement

The raw data supporting the conclusions of this article will be made available by the authors, without undue reservation.

## Ethics statement

The studies involving humans were approved by Comitato Etico della Brianza, Via Pergolesi 33 - Monza (Italy). The studies were conducted in accordance with the local legislation and institutional requirements. Written informed consent for participation in this study was provided by the participants’ legal guardians/next of kin.

## Author contributions

AC: Conceptualization, Investigation, Methodology, Supervision, Validation, Writing – original draft, Writing – review & editing, Data curation. GRu: Supervision, Validation, Writing – review & editing. GC: Data curation, Formal analysis, Software, Writing – original draft, Writing – review & editing. GRo: Data curation, Validation, Writing – review & editing. MN: Data curation, Validation, Writing – original draft. SM: Data curation, Validation, Writing – original draft. DT: Data curation, Formal analysis, Software, Writing – original draft. CP: Validation, Writing – review & editing. SR: Data curation, Writing – review & editing. AA: Data curation, Writing – original draft. KF: Data curation, Writing – review & editing. GT: Data curation, Writing – review & editing. PP: Validation, Writing – review & editing. ABo: Validation, Writing – review & editing. CG: Validation, Writing – review & editing. SLCM: Validation, Writing – review & editing. MS: Validation, Writing – review & editing. EG: Validation, Writing – review & editing. ABi: Supervision, Validation, Writing – review & editing. ABa: Supervision, Validation, Writing – review & editing. CB: Supervision, Validation, Writing – review & editing.
